# Stress during Adolescence Alters Palatable Food Consumption in a Context-Dependent Manner

**DOI:** 10.1371/journal.pone.0148261

**Published:** 2016-02-12

**Authors:** Christine Handy, Stephanie Yanaga, Avery Reiss, Nicole Zona, Emily Robinson, Katherine B. Saxton

**Affiliations:** Department of Biology, Santa Clara University, Santa Clara, California, United States of America; University of Leicester, UNITED KINGDOM

## Abstract

Food consumption and preferences may be shaped by exposure to stressful environments during sensitive periods in development, and even small changes in consumption can have important effects on long term health. Adolescence is increasingly recognized as a sensitive period, in which adverse experiences can alter development, but the specific programming effects that may occur during adolescence remain incompletely understood. The current study seeks to explore the effects of stress during late adolescence on consumption of a palatable, high-fat, high-sugar food in adulthood—under basal conditions, as well following acute stress. Male Long-Evans rats were exposed to a regimen of variable stress for seven days in late adolescence (PND 45–51). During the stress regimen, stressed animals gained significantly less weight than control animals, but weight in adulthood was unaffected by adolescent stress. Palatable food consumption differed between experimental groups, and the direction of effect depended on context; stressed rats ate significantly more palatable food than controls upon first exposure, but ate less following an acute stressor. Leptin levels and exploratory behaviors did not differ between stressed and non-stressed groups, suggesting that other factors regulate preference for a palatable food. Altered food consumption following adolescent stress suggests that rats remain sensitive to stress during late adolescence, and that adult feeding behavior may be affected by previous adverse experiences. Such programming effects highlight adolescence as a period of plasticity, with the potential to shape long term food consumption patterns and preferences.

## Introduction

Environmental influences on metabolic function and obesity have garnered much recent attention, coinciding with the growing obesity epidemic. The multitude of factors influencing food choices range from individual life history factors to the physical and social environment [1]. In addition, recent research posits that even small changes in calorie consumption and food choice can have large effects on obesity risk [2]. Early-life experiences can alter risk of disease by increasing vulnerability or resilience, depending on environmental circumstances. While some descriptions of developmental plasticity focus on the enduring damage from adverse early life experiences [3], others suggest that developing organisms undergo adaptations to better meet the challenges of the predicted environment [4]. Theory and research also suggest that stress during early life, including both gestation and the early post-natal period, may increase sensitivity to later environmental contexts, whether positive or negative [5]. Early life stress has been associated with adulthood food intake [6] and obesity [7], and stress during certain critical windows in development can result in lasting—and even permanent—changes in metabolic function [8–11]. Although less thoroughly studied than early life, adolescence has been proposed as an additional sensitive period relevant to eating behavior and metabolic function [12–16].

Adolescence is a time of physiological, neuroendocrine, and behavioral changes and serves as a transition between childhood and adulthood. This sensitive period for brain development includes maturation of stress-related systems and structures such as the hypothalamic-pituitary-adrenal (HPA) axis, mesolimbic dopamine system, and the prefrontal cortex [14–16]. Adolescence is characterized by overproduction of axons and synapses, followed by dendritic pruning in the amygdala, nucleus accumbens, and prefrontal cortex, as well as a peak in dopamine signaling [17]. The hippocampus continues to develop into adulthood, and the HPA axis response to stress differs between adolescents and adults [14, 18, 19], with increased exposure to glucocorticoids resulting from stress during adolescence [15]. The continued development and plasticity of stress-sensitive brain regions suggests that stress during adolescence can affect brain development and exert programming effects on both biology and behavior into adulthood [14, 15].

In humans, stress during adolescence has been associated with increased risky decision making [20, 21], and decreased self-control [22]. Research has also identified a positive relationship between perceived stress and unhealthy eating patterns [23, 24]. However, the links between stress in adolescence and behavior in adulthood have not yet been fully elucidated in human populations. In laboratory rat models, stress during adolescence has been shown to affect exploratory, novelty-seeking, and risk-taking behaviors, although the direction of the effect varies, depending on timing, type, and severity of stress exposure. For example, stress during early adolescence led to a decrease, whereas stress during mid-adolescence led to increased, open arm exploration in the elevated plus maze [25]. Similarly, rats exposed to social adversity in early adolescence displayed increased proactive coping in response to stress, whereas adults showed reduced proactive coping in the short term [26]. Rats stressed during mid-adolescence and rats exposed to a non-social stressor showed no behavioral differences [26]. Furthermore, psychogenic stress before and during puberty (PND28-42) has been associated with increased risk-taking and novelty-seeking behaviors in late adolescence, as well as increased food intake following forced swimming [27]. Thus, adolescent stress may increase curiosity or decrease anxiety, in contrast to stress earlier in life, which is often considered anxiogenic [28, 29].

Although, to our knowledge, no studies have yet examined adolescence as a critical period for the formation of food preferences [30], stress exposure has been linked to alterations in food consumption. Stress induces both over and under-eating in humans and animals, which may be due to the nature, severity, and duration of the stressor [31, 32]. In both human and animal studies, food consumption shifts toward selection of highly palatable, high-fat, and high-sugar foods in response to acute stress, if those foods are available [33], regardless of whether the overall number of calories consumed increases or decreases [34, 35]. Stress-induced eating has been hypothesized to activate the brain reward system and decrease the activity of the HPA axis by means of opioid release [32], and consumption of palatable food leads to negative feedback of the HPA axis, mimicking the process seen in stress recovery [33, 36]. Long term, such changes can result in an increased risk for obesity [35]. Although mechanistic links between stress, eating behavior, and obesity are not fully understood, leptin has been repeatedly suggested as an important biomarker in the relationship between stress and weight gain [33, 37–39].

The current study examines the effects of variable stress (restraint, fox odor, and tail pinch) during late adolescence on consumption of a palatable high-fat, high-sugar food in adulthood, both under basal conditions and after acute stress, among male Long-Evans rats. We hypothesize that stress during adolescence will affect adult behavior in a context-dependent manner. We also investigate the effects of adolescent stress on exploratory behavior and leptin levels in adulthood, as potential mediators of the relationship between stress and food consumption.

## Materials and Methods

### Animals

Thirty-two, juvenile male Long-Evans rats, obtained from Charles River (Hollister, CA) on PND 23, were pair housed in 48 x 27 x 20 cm polycarbonate cages on TEK-Fresh laboratory bedding (Harlan Teklad, Madison,WI). Rats were housed on a 12h:12h light/dark schedule with lights on at 7:00am. Rats had constant access to Global 18% protein rodent diet (Harlan Teklad, Madison, WI) and water. Rats were weighed at least two times per week throughout the study. The data from one cage were excluded from all analyses, due to greater than 10% decrease in body weight of one individual during the study. All procedures were reviewed by the Santa Clara University Institutional Animal Care and Use Committee (protocol #A4443-01) and conformed to NIH guidelines for the care and use of laboratory animals. All efforts were made to minimize animal suffering and to reduce the number of animals used.

### Adolescent Stress

Rats were randomly assigned to the stress and control groups (n = 16 in each group). The stress group was exposed to variable stressors for seven consecutive days from PND 45–51, in a separate room from the control rats. PND 45–51 is consistent with the post-puberty, late adolescence period in rats [27, 40]. Rats were weighed daily during the stress regimen. Three techniques were used to stress the experimental group during adolescence: restraint, exposure to fox odor, and tail pinch stress. A single stressor was applied each day for seven consecutive days, at a random time during the light period to minimize habituation and predictability. All animals in the stress group were removed from the housing room for stress procedures at the same time, while the control group remained undisturbed in the housing room. Control animals were weighed, but otherwise left undisturbed during the stress regimen.

Restraint: Rats were immobilized in tapered plastic cones for a 15 min. period and then released. While restrained, the rats remained in their home cages.

Fox odor: A cotton ball soaked with fox urine (Wildlife Research, Ramsey, MN) was placed in each home cage and removed after 10 minutes.

Tail pinch: A wooden clothespin was placed on the rat’s tail for 10 minutes. A new clothes-pin was used for each rat and rats remained in their home cage during the test.

### Behavioral Testing

Light-dark Box: The apparatus consisted of two compartments (each: 40 x 40 x 20 cm, connected by a 10 x 10 cm opening). The dark side was made of opaque, black acrylic with a cover of the same material; the light side was transparent acrylic with no cover. Rats were placed in the dark side of the apparatus and left to explore for 5 minutes in the absence of the experimenters. The apparatus was cleaned between animals. The test was videotaped and scored at a later time by a researcher blinded to group assignment. Time to emerge from the dark compartment, time spent in light, and number of stretch postures (fewer than four paws in the light box) were scored [41]. The light-dark box test was performed prior to adolescent stress, immediately after stress, and in adulthood, at PND 33, 53, and 102 respectively.

Food Consumption: For five consecutive days, beginning at PND 127, rats were provided with a vanilla flavored, high carbohydrate, high protein milkshake (Ensure) for one hour in their home cage. Milkshakes were provided in bottles identical to the water bottles used. Bottles were weighed before and after being provided to the rats to assess the amount, in grams, consumed per cage. Throughout the food preference task, standard rat chow remained available.

The first three days allowed the rats to habituate to the presence of the milkshake. On the fourth day the rats were stressed by restraint for 15 minutes and then immediately provided with the milkshake for one hour after release. The fifth day served as the recovery day on which the rats were given the milkshake for one hour and the amount consumed was recorded. Both rats remained in their home cage during the test. We measured the total grams of milkshake consumed per cage and calculated the average consumption per rat. The unit of analysis for this task was the cage, as individual consumption was not measured.

Behavioral assays were conducted in standard lighting conditions during the light phase.

### Sacrifice

At the termination of the study all animals were sacrificed between 10am and 2pm. Rats were exposed to CO_2_ gas in their home cage, followed by decapitation. Prior to decapitation, measurements of weight (g) and body length (tip of nose to base of tail, cm) were taken to calculate BMI (weight/length^2^). Animals were dissected; trunk blood was collected, and organs (heart, liver, and kidneys) were weighed immediately.

### Hormone Assays

Trunk blood was collected from each animal at the time of sacrifice in heparinized tubes. Plasma was extracted via centrifugation, frozen immediately, and stored at -20°C for future use. Plasma samples were thawed, and leptin concentrations were measured using commercially available ELISA kits (R&D systems, Minneapolis, MN). Each sample was run in duplicate according to kit instructions. Intra-assay variation was 9.02%.

### Statistical Analysis

All analyses were conducted using Stata12 (College Station, TX). We compared weight at PND 23, 45, and 51, as well as weight gain during the stress period; for these analyses, experimental group means were compared using t-tests. Because of the repeated weight measures throughout the study, we then used longitudinal regression with robust standard errors, allowing for random effects (xtreg), to examine weight gain over time. Analysis was repeated starting at the beginning of the study (PND 23), before stress (PND 45), after stress (PND 51), and in adulthood (PND 90). T-tests were also used to compare organ weights (unadjusted and adjusted for body weight) and body mass index (BMI) at the conclusion of the study.

Data for the food preference task were analyzed using longitudinal regression with robust standard errors, allowing for random effects. Variables in both the unadjusted and adjusted regression models included group, time, and their interaction. The adjusted model included covariates for weight and exploratory behavior (measured via the light dark box). Results remained consistent when using different days as the reference and when including light dark box behavior at each time point (PND 33, 52, and 102). Associations between leptin, BMI, and food consumption were explored using Pearson correlation coefficients.

## Results

This study explored the impact of chronic mild stress during adolescence on adult behaviors and biological markers. The programming effect of adolescent stress was examined in relation to palatable food consumption, weight gain, and leptin levels during adulthood.

### Weight gain

At the start of the study, the groups did not differ in weight, as shown in Table 1. From the beginning of the study until the start of the stress regimen, despite equivalent housing and feeding conditions, the group assigned to adolescent stress gained more weight than the control group, such that the stressed group weighed significantly more than the control group on PND45, at the beginning of the stress regimen (t = 2.3, df = 28, p = 0.029). During the seven days of stress, the stressed animals gained significantly less weight than the control animals (t = -2.63, df = 28, p = 0.014), consistent with previous research [42–44].

**Table 1:** Descriptive statistics of rats exposed to adolescent stress (PND 45–51) and controls.

	Stress (n = 14)	Control (n = 16)	
	Mean (SE)	Mean (SE)	P-value
**Weight**			
PND 23	58.09 (1.29)	55.43 (1.36)	0.17
PND 45	254.35 (4.88)	239.61 (4.19)	0.029*
PND 51	301.28 (6.76)	293.17 (5.25)	0.35
PND 90	519.91 (16.26)	498.39 (10.29)	0.26
PND 130	639.52 (22.37)	613.25 (12.68)	0.3
**Weight gain during stress regimen, PND 45–51 (g)**	46.93 (2.10)	53.56 (1.48)	0.014*
**BMI (g/cm**^**2**^**)**	1.00 (0.030)	0.97 (0.017)	0.35
**Light dark box time to emerge (sec)**			
PND 33	230.93 (30.01)	207.63 (27.42)	0.57
PND 53	218.64 (30.94)	234.06 (22.36)	0.68
PND 102	220.64 (32.50)	232.88 (30.09)	0.78
**Light dark box time in light (sec)**			
PND 33	15.93 (7.52)	15.06 (4.45)	0.92
PND 53	16.07 (7.58)	19.50 (7.54)	0.75
PND 102	23.72 (14.42)	21.06 (10.07)	0.89
**Milkshake Consumed (g)** ^a^			
Day 1	21.00 (1.32)	15.65 (0.67)	0.0024**
Day 2	31.57 (1.50)	30.01 (2.02)	0.54
Day 3	31.31 (2.03)	26.19 (3.40)	0.23
Day 4	26.06 (2.06)	33.63 (3.13)	0.073
Day 5	33.53 (1.63)	35.61 (2.40)	0.50
**Leptin (ng/mL)**	20.19 (2.65)	21.59 (2.35)+	0.7

*p<0.05,

**p<0.01

^+^n = 14

^a^Milkshake consumption measured by cage (adolescent stress group: n = 7, control group: n = 8)

**Fig 1:**
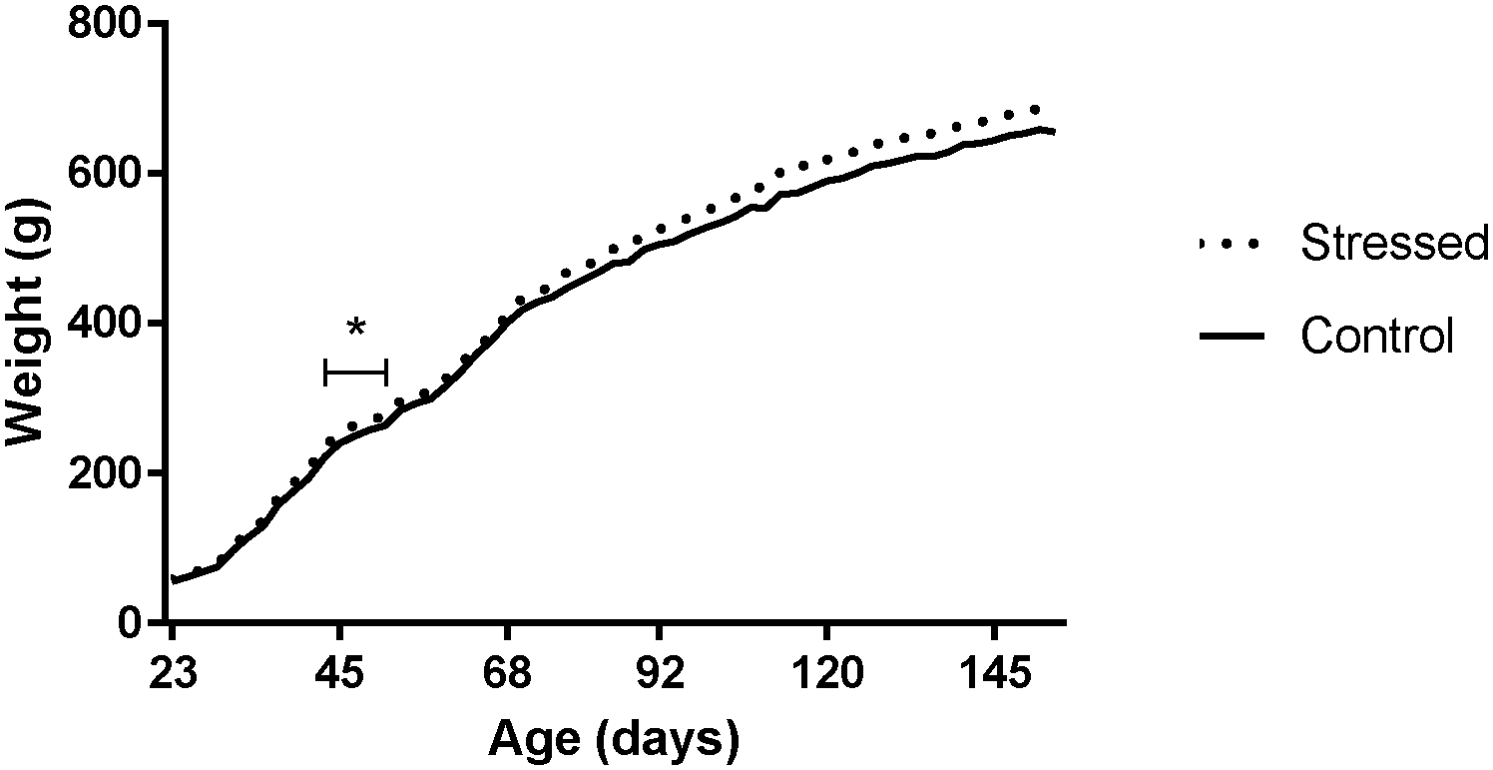
Weight gain throughout life course. The mean weight for the stressed group (dashed line, n = 14) and control group (solid line, n = 16). Weight gain differed during the stress period (PND45-51), with the stressed rats gaining significantly less weight than control rats (*p<0.05). Weight gain did not differ between groups at any time following the adolescent stress period.

There was no significant difference between the adolescent-stress rats and controls from the conclusion of stress exposure to the end of the study at PND154 (Fig 1); adolescent stress was not significantly related to weight or weight gain during adulthood. We conducted longitudinal regression using repeated measures of weight for each animal. Our model included age and stress as predictor variables. Longitudinal linear regression with robust standard errors, modeling weight from the conclusion of adolescent stress (from PND52) as a function of age (β = 8.29, p<0.01), age^2^ (β = -0.0235, p<0.01), and stress (β = 22.90, p = 0.25), demonstrated that adolescent stress was not a significant predictor of future weight, but that age was significantly related to body weight. We repeated the analysis starting at the beginning of the study (PND23), the beginning of the stress period (PND45), and at adulthood (PND90); results did not change. Thus, adolescent stress exposure did not significantly affect weight gain trajectory.

We also compared the BMI of rats at the conclusion of the study. BMI did not differ by adolescent stress experience (t = 0.95, df = 28, p = 0.35). Organ weights (liver, kidneys, and heart, adjusted for body weight) did not differ between groups at the conclusion of the study (p’s = 0.43, 0.37, 0.32 respectively).

### Food consumption

**Fig 2:**
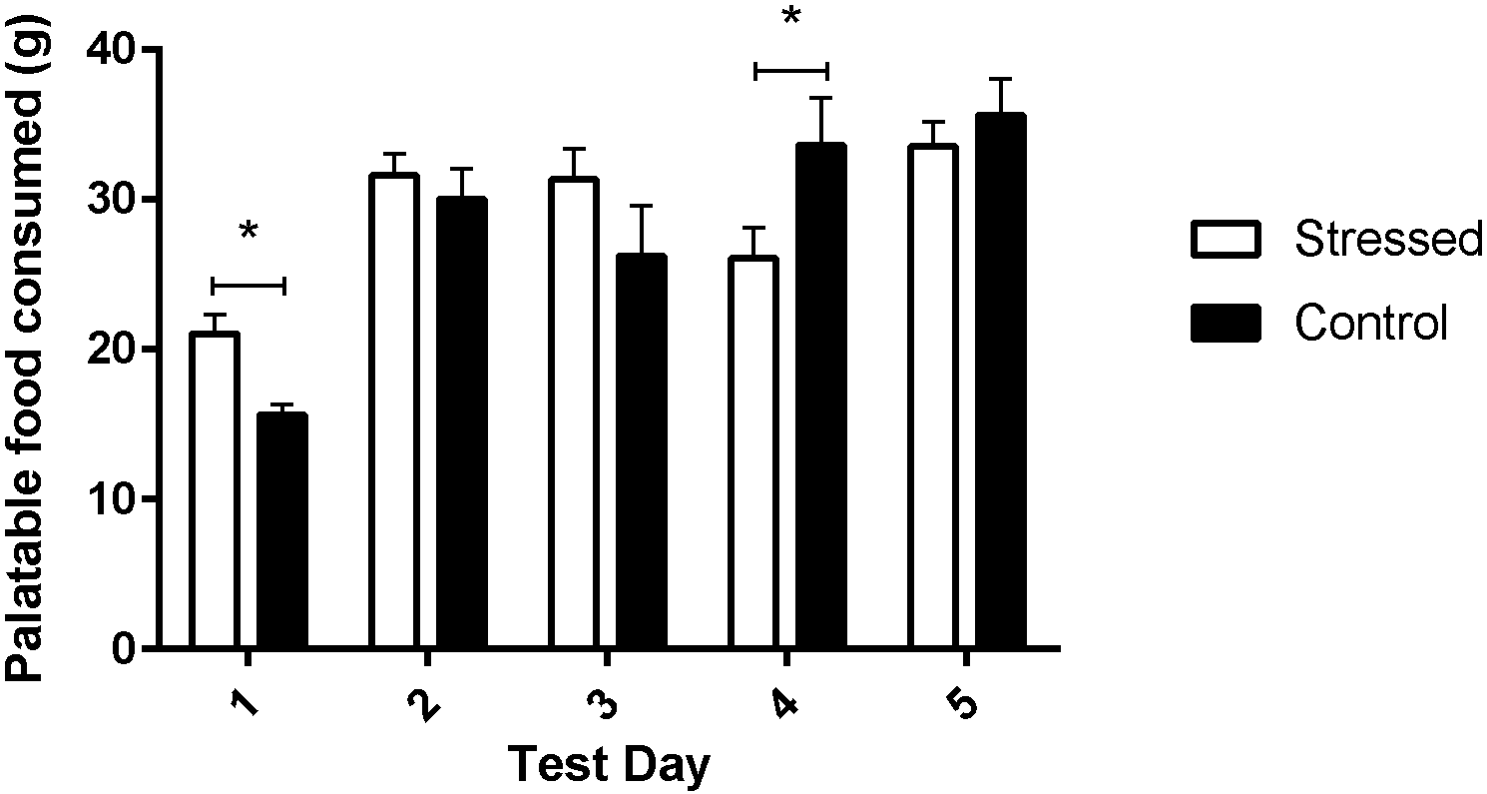
Milkshake consumption in the food preference task. The mean grams of milkshake consumed by the stressed group (black bars, n = 7) and the control group (white bars, n = 8) are shown for each day of the food preference task. Stressed rats consumed significantly more palatable food on the first day of the task, and significantly decreased consumption compared to controls on day four of the task, following acute stress (*p<0.05).

Stress during adolescence was significantly related to consumption of a palatable food in adulthood (Fig 2). Rats were presented with a high-fat, high-sugar milkshake for one hour on five consecutive days in their home cage, and consumption of the milkshake was measured as the average consumption per rat.

Rats consumed, on average, 18.14g of the milkshake on day 1, and 30.79g, 28.58g, 30.09g, 34.64g on days 2–5 respectively. Adult male rats consume approximately 30g of standard rat chow per day, equivalent to approximately 93 kcal. By comparison, rats consumed, on average, between 18.14 kcal and 34.64 kcal of the milkshake per day during the food preference task (Ensure milkshake provides 1kcal/g), or up to 38.4% of their daily average calorie intake. The milkshake, while highly palatable, is less energy dense than standard rat chow, and provides a higher proportion of calories from carbohydrates and fat, and a lower proportion from protein, compared to the standard rat chow provided throughout the study (Harlan Teklad 2018).

Adolescent stress experience significantly predicted the pattern of palatable food consumption in adulthood in a context-dependent manner. Palatable food consumption was analyzed using longitudinal regression with main effects of group and day (1–5), as well as the interaction between group and day, as shown in Table 2. Each day was entered into the model as a dummy variable, to detect different effects of each day, without assuming linearity. We used consumption on day 2 as the reference, so we could examine the interaction between day and group for the conditions of interest (day 1 and day 4). Results remained consistent when other days were used as the reference value in the regression models.

**Table 2:** Repeated measures regression models predicting consumption of palatable food (g).

	Model 1	Model 2
	β (95%CI)	β (95%CI)
Constant	30.77 (28.28, 33.25)**	23.49 (10.67, 36.31)*
Day 1	-15.03 (-17.22, -12.85)**	-14.84 (-17.21, -12.48)**
Day 2 (reference)	-	-
Day 3	-4.49 (-11.22, 2.23)	-4.31 (-11.06, 2.44)
Day 4^a^	2.94 (-3.11, 8.99)	3.13 (-3.04, 9.30)
Day 5	4.93 (0.87, 8.90)*	5.12 (1.04, 9.19)**
Day 1 * Adol. stress	5.17 (2.18, 8.16)**	4.80 (1.59, 7.80)*
Day 2 * Adol. stress	-	-
Day 3 * Adol. stress	4.94 (-2.81, 12.70)	4.57 (-3.00, 12.15)
Day 4^a^ * Adol. stress	-7.75 (-15.17, -0.33)*	-8.12 (-15.76, -0.49) *
Day 5 * Adol. stress	-2.27 (-7.91, 3.38)	-2.64 (-8.04, 2.77)
Weight PND125	-	0.011 (-0.0082, 0.031)
Light dark box, time in light, PND102	-	0.016 (-0.026, 0.0581)

Coefficients, 95% CIs, and p-values for both the unadjusted (Model 1) and adjusted (Model 2) regression models are shown. For this analysis, weight was measured on the first day of the task; exploratory behavior was measured as time in light portion of light dark box in adulthood (PND102).

^a^Acute stress exposure

*p<0.05,

**p<0.01

The adolescent stress group consumed more of the palatable food than controls on the first day, and less on the fourth day, following acute stress, as shown in Fig 2. On the first day of the food consumption task, stressed rats consumed more of the novel palatable food than the non-stressed rats (model 1, longitudinal regression: β = 5.17, z = 3.39), Table 2. Following acute restraint stress on day 4 of the food preference task, the rats that had been stressed during adolescence showed decreased palatable food consumption, compared to the control animals, as adolescent-stressed animals ate less milkshake than the control group (model 1, longitudinal regression: β = -7.75, z = 2.05).

We next explored whether the association between adolescent stress and palatable food consumption could be explained either by bodyweight or exploratory behavior (Table 2). Because bodyweight may be associated with caloric needs and food consumption patterns, we examined the relationship between weight and food consumption in the longitudinal regression model. Adjusting for bodyweight did not change the results, and body weight did not predict food consumption. Because of the novelty involved in approaching and consuming a new food item, we next examined whether differences in exploratory behavior between experimental groups might explain the observed differences in food preference. Adjusting for exploratory behavior in the light dark box on PND 33 (juvenile), 53 (following stress period), or 102 (adult), did not change the relationship between adolescent stress and amount of milkshake consumed. We repeated this analysis using either time to emerge or time in light measurements of exploratory behavior; none of these analyses changed the results.

A history of adolescent stress predicted increased milkshake consumption upon first exposure, and reduced milkshake consumption following acute stress in adulthood, independent of bodyweight and exploratory behavior. As shown in Table 2, when both weight and exploratory behavior were included in the regression model, adolescent stress and day remained the only significant predictors of food consumption. Thus, adolescent stress was associated with consumption of a high fat, high sugar food, with the direction of effect dependent on testing conditions (i.e. basal or following acute stress), independent of weight or exploratory behavior.

### Leptin

We measured plasma leptin levels in adulthood (PND 154) to further explore the effects of adolescent stress on weight gain and related endocrine parameters. Adolescent stress did not significantly predict leptin levels in adulthood (t = -0.39, df = 26, p = 0.70). Leptin was not significantly related to either adult weight or BMI overall or in either experimental group (p’s>0.39). Leptin levels did not correlate with food preference task results (p’s>0.26), and leptin did not explain the difference in behavior observed in the food preference task between experimental groups when added to the regression model.

## Discussion

We found that chronic variable stress in adolescence predicted food preference for a palatable food item in adulthood, increasing consumption upon first exposure, but decreasing consumption following acute stress. Our findings suggest a programming of food preference in adulthood by adolescent stress. The observed context-dependent phenotypic plasticity does not appear related to body mass, leptin signaling, or exploratory behavior, as none of these measures differed between adolescent-stressed and control animals. These findings may support a shift in preference or motivation, rather than a difference in caloric need—a difference in hedonic rather than homeostatic processes—as the milkshake was palatable, but an inefficient form of calorie consumption (less energy dense than rat chow), rat chow was available *ad libitum* throughout the study, and body weight was not correlated with consumption.

Although stress exposure reduced weight gain during the stress regimen, groups did not differ in patterns of weight gain in adulthood. These results are consistent with previous findings that stressed rats gain less weight during the stress period compared to controls [45, 46], and that younger rats show an increased ability to recover from temporary weight loss, compared to animals stressed as adults [47].

The current study found no relationships between stress and anxiety-related behavior in the light dark box, or between food consumption patterns and light-dark box behavior. However, we did see a difference in neophagia, as evidenced by food preference on the first day of novel food availability. These results suggest important and nuanced differences between novelty-seeking, harm-avoidance, risk-taking, and sensation-seeking behaviors. Novel food consumption goes beyond simple exploratory behavior, because there is some risk involved in consuming an unknown substance. The increases in risk taking and novelty seeking that we observed on the first day of the task may indicate specific adaptive behavioral changes to an environment characterized by unpredictable stress [48]. Future experiments should include additional behavioral tasks to further distinguish between the effects of stress during sensitive periods on the related but distinct behaviors of harm avoidance, risk avoidance, and reward seeking, to develop a more nuanced understanding of these related but distinct behavioral profiles.

Our results add to the literature emphasizing that stress-induced changes in novelty-seeking and exploratory behavior depend on the timing and severity of the stress [25, 49]. Increased novelty-seeking has been tied to early life stress in monkeys [50] and rats [51], and stress later in adolescence appears to increase exploratory behavior [25, 49]. Physical stress has been found to cause long-term decreases in preference for saccharine and in exploratory behavior in an open field, while emotional stress resulted in an increase in both areas [52]. Studies on adult rats have shown that acute restraint stress leads to decreased appetite following acute stress [47, 53], similar to the response we observed in the stressed group, while chronic stress may increase intake of palatable food [54].

Conflicting findings may reflect that the behavioral effects of stress during development depend on the threat level of the testing environment. For example, stress during adolescence improved foraging effectiveness of rats under threat conditions, including lower latency to begin foraging, increased activity, and more food consumed, while minimal differences were observed under low-threat conditions [55]. This sensitivity to context parallels our findings that the behavioral programming effects of adolescent stress depend on the environmental context in which measurement occurs.

Our study included several important limitations. We only examined male rats. Comparative studies have shown differences between male and female rats, and future studies should continue to investigate these differences [14, 56]. Because testing of food consumption took place in the home cage, we were unable to measure individual consumption. Although we found differences at the cage level, variables such as social dominance may also affect behavior.

An alternative explanation for the observed differences in food consumption could be unmeasured differences in metabolism. It is possible that rats replaced their water intake with the milkshake, thus shifting the composition of their diet. Although there were no differences in weight gain, and body weight was not associated with palatable food consumption, it is possible that unmeasured metabolic differences explain the findings. Future research should quantify both chow and water intake to further explore the mechanism behind the observed differences. Additionally, we did not measure corticosterone or catecholamine levels. Both glucocorticoids and catecholamines have been linked to feeding behavior and metabolism.

Similarly, we only measured leptin at the conclusion of the study, thus preventing the examination of leptin levels throughout the lifespan. Although our focus was the long-term effects of adolescent stress, this limitation prevents us from identifying potential short-term alterations in leptin following stress. Research in humans and animals suggests that stress-related changes in food intake may be linked to leptin signaling. In women, following acute stress, an increase in leptin levels is negatively related to the consumption of sweet, fatty foods, or “comfort foods” [57] and maternal separation in young rats has been linked to decreased leptin levels [58]. Leptin resistance is emerging as an important factor in obesity, so a greater understanding of the relationship between stress and leptin will help to elucidate leptin’s connection to obesity. Future studies could further explore the links between stress exposure during adolescence, stress hormones, behavior, and weight gain.

The relatively mild and short duration of stress exposure in this study may have limited our ability to detect differences between groups. The current protocol allowed us to isolate the effects of stress exclusively during late adolescence, defined as PND 45–51 [27], as stress can continue to affect developing animals through young adulthood [59]. Longer stress exposure per day as well as more days of stress exposure might have led to more dramatic differences, but this study was not designed to focus on the effects of severe stress. Habituation to stress and/or the stress-ameliorating effects of group housing may also have lessened the results. However, we note that even the relatively mild stress used in this study altered behavior.

Often, stress during development is described as solely damaging or fundamentally harmful. Our results, in contrast, demonstrates the complex relationship between stress and food intake and support the theory of biologic sensitivity to context [5], as animals with a history of stress exposure showed altered behavior in both directions compared to controls, dependent on context. The long-term effects of such behavioral differences, in the context of continued availability of a high-fat, high-sugar food, remain to be seen. It is possible that small increases in preference for such foods would lead to obesity among individuals with a history of adolescent stress if those foods were continually available under low stress conditions, but not if the environment is stressful. In the current study, animals exposed to stress during adolescence were more sensitive to their later life context, suggesting adolescence may provide a window of opportunity for nutrition-related interventions. For example, preventing stress during adolescence, providing coping outlets, or limiting access to novel palatable foods may alter food preference and consumption in adulthood.

Expanding the developmental mismatch theory from early life [60], adolescence constitutes a sensitive period during which programming can occur, with the goal of adjusting the phenotype to match the expected adult environment [16, 55]. Thus, adolescent stress may increase risk under low-threat conditions in adulthood, but lower risk in more stressful environments.
